# Sortilin and atherosclerosis

**DOI:** 10.18632/oncotarget.5098

**Published:** 2015-08-04

**Authors:** Martin B. Mortensen, Mads Kjolby, Jacob F. Bentzon

**Affiliations:** Department of Clinical Medicine, Aarhus University; and Department of Cardiology, Aarhus University Hospital Denmark, Aarhus, Denmark

The complexity and intricacy of nature has recently been illustrated with the role of sortilin in atherosclerosis. Sortilin, a VPS10p sorting receptor initially identified in human brain tissue, suddenly gained much attention from the cardiovascular community as unbiased genome-wide association studies (GWASs) repeatedly demonstrated that single nucleotide polymorphisms (SNPs) at the 1p13.3 locus was strongly associated with the risk of coronary artery disease and myocardial infarction, as well as with the level of low-density lipoprotein (LDL)-cholesterol and the development of abdominal aortic aneurism. The 1p13.3 region harbors four genes *CELSR2*, *PSRC1*, *MYBPHL* and *SORT1.* The *SORT1* gene, encoding sortilin, was soon implicated as the causative gene and studies on sortilin and atherosclerosis initially focused on the role of sortilin in hepatic lipoprotein metabolism. In a seminal paper, Musunuru et al. [1] found that one of the most strongly associated SNPs at 1p13.3 changes a binding site for the transcription factor C/EBFα and affects sortilin expression levels in human hepatocytes. However, pinpointing the underlying molecular mechanisms of sortilin has proven challenging. Kjolby et al. [2] found that sortilin binds apoB100 in the Golgi and facilitates the secretion of apoB100-, but not apoB48-containing, lipoproteins from hepatocytes, resulting in a positive relationship between hepatic sortilin expression and plasma LDL levels. Strong et al. [3] also showed binding of apoB100 to sortilin, but found that binding resulted in trafficking to lysosomes and a negative relationship between hepatic sortilin expression and plasma LDL levels. More recently, Gustafsen et al. [4] reported that sortilin facilitates PCSK9 secretion from hepatocytes, which in turn may increase LDL levels by down-regulating LDL receptors. Thus, the functional role of sortilin in hepatic lipoprotein metabolism is likely multiplex, involving several mechanisms, which dependent on the context may increase or decrease the level of circulating LDL [5]. Genetic studies in humans indicate that decreased hepatic sortilin expression is associated with an average net increase in LDL cholesterol and in the incidence of atherosclerotic cardiovascular disease [1].

In a recent study [6] we uncovered more complexity in the involvement of sortilin in atherosclerosis. Using the *Apoe*^−/−^ mouse we demonstrated that sortilin also directly affects atherogenesis, independent of its regulatory role in lipoprotein metabolism. In the *Apoe*^−/−^ mouse, the major defect is in the clearance of apoB48-containing lipoproteins that are normally removed by apoE-mediated binding to LRP1 or LDLR. Consequently, the vast majority of atherogenic lipoproteins in this setting are of the apoB48-containing type, which sortilin does not bind [2], and indeed, we found no or minimal differences in total plasma cholesterol or lipoprotein size profiles fractions between *Sort1*^−/−^ and *Sort1*^+/+^ mice on the *Apoe*^−/−^ background. This enabled us to uncover a direct effect of sortilin on atherogenesis. First, we showed that global deficiency of sortilin in *Apoe*^−/−^ mice reduced the development of both early and late atherosclerotic lesions. Second, we identified sortilin as a selective high-affinity sorting receptor for the proinflammatory cytokines interleukin-6 and interferon-γ. Consistently, we found that mice with a sortilin deficient bone marrow had decreased development of atherosclerosis and reduced systemic markers of inflammation, indicating that sortilin expression in immune cells directly promotes the development of atherosclerosis in the vascular wall by facilitating inflammation. In an independent study published soon after, Patel et al. [7] confirmed the pro-atherosclerotic effects of sortilin in immune cells using *Ldlr*^−/−^ mice with a study design very similar to ours, but concluded that the underlying mechanism was reduced uptake of LDL by sortilin-deficient macrophages. In our experiments, which included similar but not exactly the same assays, we did not observe such an effect. The reason for this discrepancy is not known. It was suggested by Patel et al. that the fluorescence labeling technique used in our experiments might interfere with the sortilin-apoB100 interaction. We have tested for this possibility but found that fluorescently-labeled LDL and unlabeled LDL demonstrate equally high binding affinity to LDLR and sortilin (unpublished surface plasmon resonance data). Taking both studies at face value, they demonstrate a strong pro-atherosclerotic effect of sortilin in circulating immune cells, and indicate that multiple pathways in macrophages and lymphocytes may contribute.

What has the experimental data taught us so far? Sortilin is a receptor with many known, as well as potentially many unknown, ligands, and it serves a number of functions without being essential to any of them. Clearly, we have learned that the role of sortilin in atherosclerosis is multifaceted involving distinct pathways in lipoprotein metabolism and in vessel wall inflammation, but the broad functional repertoire of sortilin is also reflected by the fact that it has been inculpated in a number of other diseases, including abdominal aortic aneurism, neurodegenerative and inflammatory diseases.

This constitutes a complicated drug target. As a sorting receptor for many proteins, inhibiting sortilin affects the balance between several biological pathways, some of which may be atherosclerosis-promoting and others atherosclerosis-protecting. The net result of perturbing this balance may be different between species, which makes it difficult to translate from the murine studies to effects in humans. It may also potentially differ between human subjects, because underlying genetic variation or the presence of co-morbidities may perturb the relative importance of the different pathways affected by sortilin. On the other hand, being mediated by multiple moderate effects rather than a single pathway could turn out to be a strength of sortilin-blocking strategies, potentially creating a favorable balance between efficacy and toxicity.

Clearly, the broad impact of sortilin in many biological systems warrants further research and is likely to be the subject of intense investigation in the future. Unraveling the puzzle of sortilin appears to have just begun.
